# Consolidated Biochemical Profile of Subacute Stage Traumatic Brain Injury in Early Development

**DOI:** 10.3389/fnins.2019.00431

**Published:** 2019-05-03

**Authors:** Jyothsna Chitturi, Ying Li, Vijayalakshmi Santhakumar, Sridhar S. Kannurpatti

**Affiliations:** ^1^Department of Radiology, Rutgers Biomedical and Health Sciences, New Jersey Medical School, Newark, NJ, United States; ^2^Department of Pharmacology, Physiology & Neuroscience, Rutgers Biomedical and Health Sciences, New Jersey Medical School, Newark, NJ, United States; ^3^Department of Molecular, Cell and Systems Biology, University of California, Riverside, Riverside, CA, United States

**Keywords:** traumatic brain injury, mitochondria, metabolomics, glycolysis, lipid metabolism, amino acid metabolism, pediatric, citric acid cycle

## Abstract

Traumatic brain injury (TBI) in general has varied neuropathological consequences depending upon the intensity and biomechanics of the injury. Furthermore, in pediatric TBI, intrinsic developmental changes add further complexity, necessitating a biochemical dimension for improved TBI characterization. In our earlier study investigating the subacute stage TBI metabolome (72 h post-injury) in a developmental rat model, significant ipsilateral brain biochemical changes occurred across 25 metabolite sets as determined by metabolite set enrichment analysis (MSEA). The broad metabolic perturbation was accompanied by behavioral deficits and neuronal loss across the ipsilateral hemisphere containing the injury epicenter. In order to obtain a consolidated biochemical profile of the TBI assessment, a subgrouping of the 190 identified brain metabolites was performed. Metabolites were divided into seven major subgroups: oxidative energy/mitochondrial, glycolysis/pentose phosphate pathway, fatty acid, amino acid, neurotransmitters/neuromodulators, one-carbon/folate and other metabolites. Subgroups were based on the chemical nature and association with critically altered biochemical pathways after TBI as obtained from our earlier untargeted analysis. Each metabolite subgroup extracted from the ipsilateral sham and TBI brains were modeled using multivariate partial least square discriminant analysis (PLS-DA) with the model accuracy used as a measurable index of TBI neurochemical impact. Volcano plots of each subgroup, corrected for multiple comparisons, determined the TBI neurochemical specificity. The results provide a ranked biochemical profile along with specificity of changes after developmental TBI, enabling a consolidated biochemical template for future classification of different TBI intensities and injury types in animal models.

## Introduction

Traumatic brain injury (TBI) is a major cause of long term disability worldwide with higher numbers of pediatric patients in the age range of 4–19 years (Faul et al., 2010; Brain Injury Association of America, 2014). Developmental differences in TBI outcomes can be attributed to intrinsic nature of the developing brain, presenting unique metabolism, cerebral blood flow (CBF), neurotransmission (Chiron et al., 1992; Biagi et al., 2007; Hales et al., 2014), (Chugani et al., 1987), proportionally higher cell numbers, increased central nervous system (CNS) myelination (Dobbing and Sands, 1973), (Reiss et al., 1996; Paus et al., 2001) and a constantly evolving neurovascular activity (Biagi et al., 2007; Labarthe et al., 2009; Winter et al., 2011; Hales et al., 2014), These progressive changes lead to highly variable outcomes on the already broad TBI pathology, which depends on injury intensity and biomechanics (Park et al., 2008; Ma et al., 2019), making TBI classification in animal models of injury a current challenge. Hence a biochemical dimension to characterize preclinical TBI models may help improve TBI classification and additionally provide mechanistic clues to the varied pathological outcomes.

Our prior Liquid Chromatography/Mass Spectrometry (LC-MS) based study of the TBI metabolome, characterized the ongoing subacute stage (72 h post-TBI). TBI of a mild to moderate intensity was induced in developing rats (age P31), translating to human age range of 4–7 years (Chitturi et al., 2018). A top-down untargeted metabolite set enrichment analysis (MSEA) revealed a diverse set of affected metabolite sets in at least 25 different biochemical pathways that broadly related to amino acid, fatty acid, one-carbon/folate, neurotransmitter and energy metabolism (Chitturi et al., 2018). In the current study, a reduction (or subgrouping) of the various identified metabolite constituents in the specific pathways affected by TBI was performed to biochemically consolidate the injury outcome. We hypothesized that ranking the broad biochemical pathway changes after TBI may provide a measurable index of the relative metabolic shifts in developmental TBI.

After lateral fluid percussion induced TBI in rats aged P31, brains were removed at 72 h post-TBI. Extracts from the ipsilateral hemisphere containing the injury epicenter were analyzed using LC-MS. 190 identified metabolites were subgrouped according to their relationship to oxidative energy and mitochondrial metabolism, glycolysis/pentose phosphate pathway, neurotransmitters/neuromodulator metabolism (polyamines), fatty acid metabolism, amino acid metabolism or one-carbon/folate metabolism, while the remaining were categorized as other metabolites. Metabolites showing highly significant change (determined by volcano plots), corrected for multiple comparisons, determined the TBI neurochemical “specificity.” A multivariate partial least square discriminant analysis (PLS-DA) of sham versus TBI in each sub-category was performed and the PLS-DA model accuracy defined neurochemical “impact.”

## Materials and Methods

### Animals

Male Sprague-Dawley rats (∼23-days-old; weighing 60–80 g) were procured from Charles River Laboratories, Wilmington, MA, United States. Animals were housed in pairs under controlled conditions and used for the experiments at age 31 days. All procedures were approved by the Institutional Animal Care and Use Committee of Rutgers Biomedical and Health Sciences-New Jersey Medical School and conducted in accordance with the National Institutes of Health guide for the care and use of Laboratory animals (NIH Publications No. 8023, revised 1978).

### Lateral Fluid-Percussion Injury

Animals (*n* = 16) were randomly assigned to sham and injury groups at age P31. A lateral fluid percussion injury (FPI) was performed in a similar manner as our previous studies (Chitturi et al., 2018, 2019). Briefly, rats were anesthetized with Ketamine (80 mg/kg i.p)-Xylazine (10 mg/kg i.p) and positioned on a stereotaxic frame. After ascertaining surgical plane anesthesia with absent tail-pinch reflexes, a 3 mm craniotomy was performed on the left side of the skull -5 mm posterior to the bregma and 3 mm lateral to the sagittal suture keeping the dura intact. A Luer-Lock syringe hub was glued surrounding the exposed dura using a cyanoacrylate adhesive. 24 h later, injury was induced by attaching the Luer-Lock hub to the FPI device (Virginia Commonwealth University, Richmond, VA, United States), with the rat under isoflurane anesthesia. A pendulum-drop delivered a brief 20 ms impact on the intact dura. The impact pressure was measured by an extra-cranial transducer and controlled between 1.8 and 2.0 atm. TBI group underwent the FPI procedure while sham animals were isoflurane-anesthetized and attached to the FPI device without the pendulum drop. Sham group (*n* = 6) and TBI group (*n* = 9) were further considered for the studies as mortality occurred in one TBI animal within 30 min after the FPI procedure. Animals were monitored within their cage environment daily throughout the duration of the experiments.

### Tissue Sample Preparation, Extraction and Liquid Chromatography/Mass Spectrometry (LC/MS)

Animals were decapitated and brains rapidly removed (<60 s). Cerebellum and brain stem were removed, and the cerebral hemispheres longitudinally separated. Each hemisphere was snap frozen in liquid nitrogen and stored in -80°C. Brain tissue samples were weighed and disrupted in extraction buffer (80% methanol in water) using a micro-homogenizer. Each sample was transferred to a pre-cooled (dry ice) homogenization tube and 4 ml of pre-cooled 80% methanol was added to each sample and homogenization was performed for 15 s using the standard micro homogenizer (Pro Scientific). 500 μL of sample was taken out to a new Eppendorf tube centrifuged at 4°C for 15 min at 14,000 rpm. Supernatants were collected and normalized to tissue weight.

Targeted LC/MS analyses were performed on the brain extract samples using a Q Exactive Orbitrap Hybrid Mass Spectrometer (Thermo Scientific) coupled to a Vanquish UPLC system (Thermo Scientific). The Q Exactive operated in a polarity-switching mode. A SeQuant ZIC-HILIC column; 2.1 × 150 mmi.d, (Merck. Co., Kenilworth, NJ, United States), was used for metabolite separation. Buffers consisted of HPLC buffer A (100% acetonitrile), and HPLC buffer B (pH = 9.0: 95% (vol/vol) water, 5% (vol/vol) acetonitrile, 20 mM ammonium hydroxide, 20 mM ammonium acetate). HPLC flow rate was set at 150 μL/min and gradients were from 85 to 30% for buffer A in 20 min followed by a wash with 30% buffer A and re-equilibration at 85% buffer A. Metabolites were identified by exact mass within 5 ppm and standard retention times. Relative metabolite quantification was performed based on peak area for each identified metabolite (Goncalves et al., 2018). The reliable high-resolution accurate mass determining capability of the Q Exactive LC-MS eliminated the need for technical replicates on biological samples.

### Data Analysis and Statistics

To minimize variations introduced during sample preparation effects of ion suppression, metabolite peak intensities were normalized to tissue-wet weight. Due to deviations from normality as tested by Shapiro-Wilk’s test, data were subsequently log transformed and auto-scaled (mean-centered and divided by the standard deviation of each variable) effectively converting them to *Z*-scores. These preprocessing steps mitigated any experimental/sample analysis variability in the metabolite peaks. Volcano plots were used to compare the size of the fold change to statistical significance. Volcano plots of significantly changing metabolites were determined using a two-sample Student’s *t*-test with a probability threshold of *P* < 0.05 corrected for multiple comparisons using the false discovery rate (FDR) for type-1 error control.

Pathway-based subgroups were identified based on the metabolite relationship to critical biochemical networks implicated in TBI such as oxidative energy metabolism, glycolysis/pentose phosphate pathway, neurotransmitters and neuromodulators (polyamines), fatty acid metabolism, amino acid metabolism, and one-carbon/Folate metabolism. Multivariate analysis considering several dependent variables was performed using the software Metaboanalyst 3.0^1^, (Xia et al., 2015). PLS-DA was used to determine the separation between groups of the metabolite variables through rotation of the principal components obtained by PCA. PLS-DA was used due to the presence of more metabolite variables than observations in addition to correlation among the variables in the data (Tang et al., 2014). PLS-DA analysis also was used for feature selection to generate feature importance measures (VIP: Variable Importance in Projection). *R*^2^*X* and *R*^2^*Y*, the fraction of variation that the model explains in the independent variables (*X*) and dependent variables (*Y*) and the predictive accuracy of the model (*Q*^2^*Y*), were estimated by the PLS-DA cross validation. Loading plots (top 25 variables) were used to determine the clustering of variables and testing their relationship within (weightings) for score changes in response to TBI.

## Results

In a top-down metabolic exploratory analysis as reported in our earlier studies (Chitturi et al., 2018), largest metabolite shifts were observed between sham and TBI in the ipsilateral hemisphere, where about 33% of the identified metabolites showed a two-fold change. Based on earlier top-down approach showing changes in 25 metabolite sets as determined by MSEA (Chitturi et al., 2018), the present study focused on biochemical consolidation. The 190 identified metabolites were subgrouped based on their relationship to broader biochemical pathways e.g., oxidative energy and mitochondrial metabolism, glycolysis and pentose phosphate pathway, fatty acid metabolism, amino acid metabolism, neurotransmitters/neuromodulators (polyamines) and one-carbon/folate metabolism. Remaining were subgrouped as other metabolites (Supplementary Table S1). Biochemical specificity and impact of TBI-induced changes were determined in each subgroup.

### Oxidative Energy and Mitochondrial Metabolism

**FIGURE 1:**
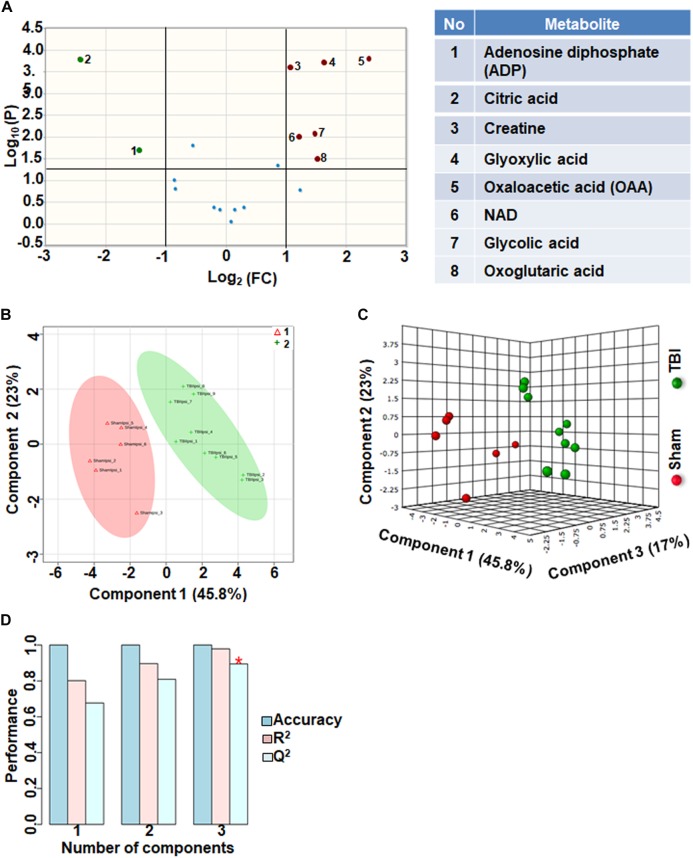
Oxidative energy metabolism at 72 h after TBI in the developing brain. **(A)** Volcano plot showing fold changes (FC) on *x*-axis and the negative logarithm (base 10) of the FDR-adjusted *P*-values on *y*-axis. Black vertical and horizontal lines reflect the filtering criteria (FC = 1.0 and FDR based corrected *P*-value < 0.05). Green or red dots represent metabolites that are significantly increased or decreased after TBI, respectively. 45% of the metabolites showed a significant change between sham and TBI. Table on right shows metabolites that are significantly different as denoted by respective numbers in the figure on the left panel. **(B,C)** PLS-DA analysis of the metabolite subgroup showing distinct class separation between sham and TBI. **(D)** PLS-DA model validation analysis and the predictive accuracy indicate high accuracy of *Q*^2^ = 0.67, 0.80, and 0.89 for the three components, respectively.

Eighteen metabolites that participate in the tricarboxylic acid (TCA) cycle, electron transport chain and mitochondrial metabolism were grouped together as oxidative energy and mitochondrial metabolites. Comparison between sham and TBI indicated significant change in 8 out of the 18 metabolites (Figure 1A). Levels of citric acid and ADP were increased in TBI animals compared to sham, whereas Oxaloacetic acid, glycolic acid, glyoxylic acid, creatine, NAD and Oxoglutaric acid decreased in TBI animals (Figure 1A). Multivariate analyses using PLS-DA distinguished between sham and TBI classes (Figure 1B,C) with a high accuracy as confirmed from the PLS-DA model parameters (Accuracy = 1.0, *R*^2^ value = 0.97, *Q*^2^ value = 0.89) (Figure 1D).

### Fatty Acid Metabolism After TBI

**FIGURE 2:**
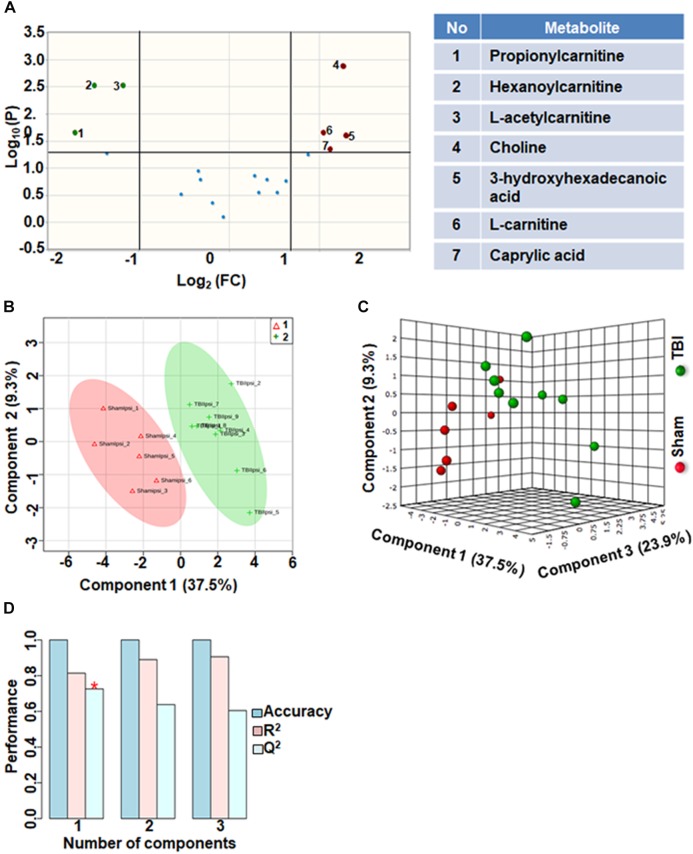
Fatty acid metabolism after TBI at 72 h after TBI in the developing brain. **(A)** Volcano plot showing FC on *x*-axis and FDR-adjusted *P*-values on *y*-axis. Black vertical and horizontal lines reflect the filtering criteria (FC = 1.0 and FDR corrected *P*-value < 0.05). Green or red dots represent metabolites that are significantly increased or decreased after TBI, respectively. 36% of the metabolites showed a significant change between sham and TBI. Table on right shows metabolites that are significantly different as denoted by respective numbers in the figure on the left panel. **(B,C)** PLS-DA analysis of the fatty acid metabolite subgroup showing distinct class separation between sham and TBI. **(D)** PLS-DA model validation analysis and the predictive accuracy indicate good accuracy of *Q*^2^ = 0.72, 0.63 and 0.60 for the three components, respectively.

Comparison of fatty acid metabolites between sham and TBI showed significant differences in 7 metabolites (36%) out of 19-targeted metabolites (Figure 2A). Choline, an important biochemical component of membrane phospholipids decreased after TBI, suggesting decreased membrane turnover and neuronal viability (Sanders and Zeisel, 2007). L-carnitine, caprylic acid and 3-hydroxyhexadecanoic acid were also decreased as a result of TBI. TBI also resulted in increased levels of acylcarnitines including propionylcarnitine, hexanoylcarnitine and L-acetylcarnitine (Figure 2A). Multivariate analyses using PLS-DA (Figure 2B,C) showed distinct class separation between sham and TBI with a good accuracy of the PLS-DA model (Accuracy = 1.0, *R*^2^ value = 0.81, *Q*^2^ value = 0.72) (Figure 2D).

### TBI-Induced Alterations in One Carbon/Folate Metabolism

**FIGURE 3:**
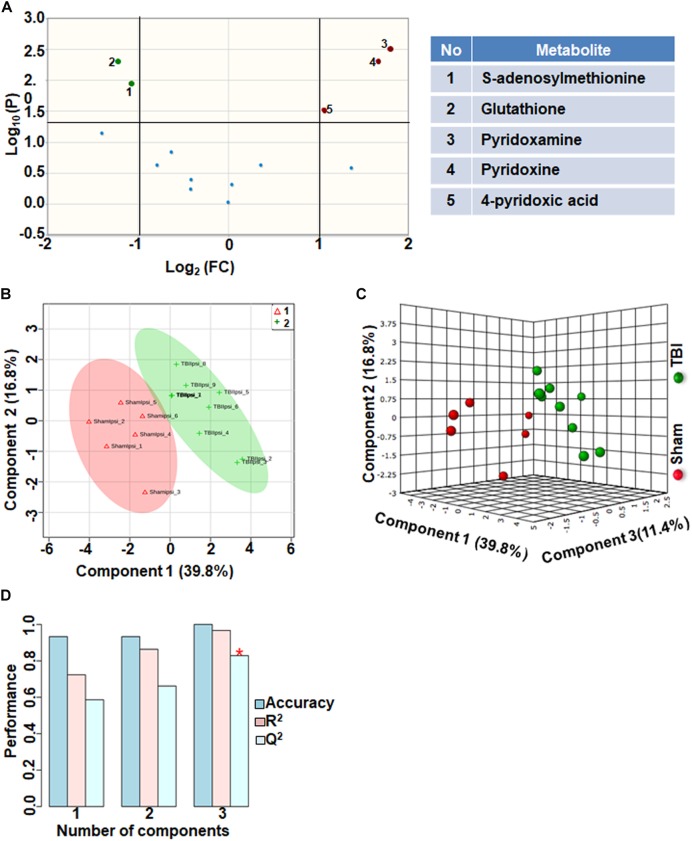
One carbon/folate metabolism at 72 h after TBI in the developing brain. **(A)** Volcano plot showing FC on *x*-axis and FDR-adjusted *P*-values on *y*-axis. Black vertical and horizontal lines reflect the filtering criteria (FC = 1.0 and FDR corrected *P*-value < 0.05). Green or red dots represent metabolites that are significantly increased or decreased after TBI, respectively. 35% of the metabolites showed a significant change between sham and TBI. Table on right shows metabolites that are significantly different as denoted by respective numbers in the figure on the left panel. **(B,C)** PLS-DA analysis of the carbon/folate metabolism subgroup showing distinct class separation between sham and TBI. **(D)** PLS-DA model validation analysis and the predictive accuracy indicate high accuracy of *Q*^2^ = 0.58, 0.66 and 0.82 for the three components, respectively.

Comparison of metabolites that participate in one-carbon metabolism between sham and TBI showed significant differences in 5 metabolites (35%) out of the 14-targeted metabolites (Figure 3A). Levels of S-adenosylmethionine (SAMe) and glutathione increased after TBI, whereas levels of pyridoxamine (vitamin B6), pyridoxine (vitamin B6) and 4-pyridoxic acid were significantly reduced (Figure 3A). Multivariate analyses using PLS-DA (Figure 3B,C) showed distinct class separation between sham and TBI with a high accuracy of the PLS-DA model (Accuracy = 1.0, *R*^2^ value = 0.96, *Q*^2^ value = 0.82) (Figure 3D).

### Glycolysis and Pentose Phosphate Pathway

**FIGURE 4:**
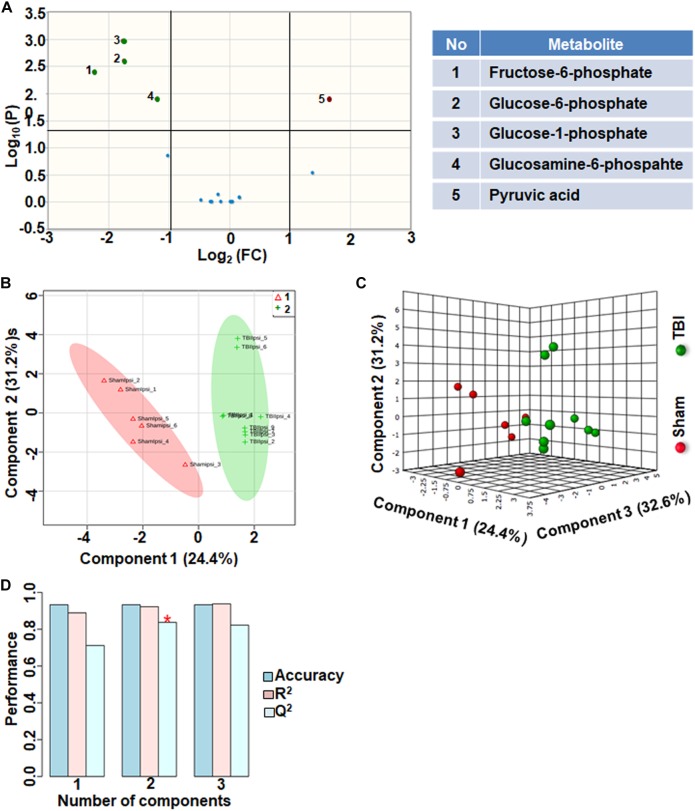
Glycolysis and pentose phosphate metabolism 72 h after TBI in the developing brain. **(A)** Volcano plot showing FC on *x*-axis and FDR-adjusted *P*-values on *y*-axis. Black vertical and horizontal lines reflect the filtering criteria (FC = 1.0 and FDR corrected *P*-value < 0.05). Green or red dots represent metabolites that are significantly increased or decreased after TBI, respectively. 30% of the metabolites showed a significant change between sham and TBI. Table on right shows metabolites that are significantly different as denoted by respective numbers in the figure on the left panel. **(B,C)** PLS-DA analysis of the metabolite subgroup showing distinct class separation between sham and TBI. **(D)** PLS-DA Model validation analysis and the predictive accuracy indicate high accuracy of Q^2^ = 0.71, 0.83 and 0.82 for the three components, respectively.

Citrate increased significantly after TBI with a high fold change. However, citrate is a negative allosteric inhibitor of the glycolytic enzyme phosphofructokinase, and its increase can slow down glycolytic breakdown of glucose into pyruvate (Garland et al., 1963). Therefore, we analyzed the subgroup containing 17 glycolysis and pentose phosphate pathway (PPP) metabolites. 30% of the metabolites showed significant changes in the glycolysis and PPP subgroup (Figure 4A). TBI-induced changes included increased levels of glucose 1-phosphate, glucose 6-phosphate, glucosamine 6-phosphate and fructose 6-phosphate (Figure 4A). Consistent with the inhibitory role of citrate on glycolysis, levels of pyruvate, the end product of glycolysis, decreased after TBI (Figure 4A). Multivariate analyses using PLS-DA showed distinct class separation between sham and TBI (Figure 4B,C), with a high accuracy of the PLS-DA model (Accuracy = 0.93, *R*^2^ value = 0.93, *Q*^2^ value = 0.83), (Figure 4D).

### Neurotransmitters Neuromodulators and Polyamine Biosynthesis

**FIGURE 5:**
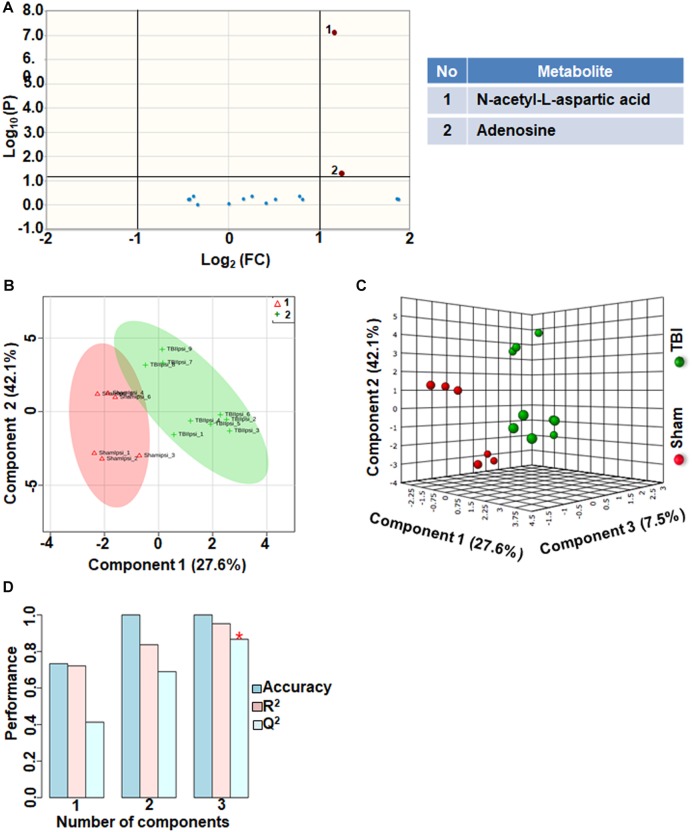
Neurotransmitters, neuromodulators and polyamine biosynthesis at 72 h after TBI in the developing brain. **(A)** Volcano plot showing FC on *x*-axis and FDR-adjusted *P*-values on y-axis. Black vertical and horizontal lines reflect the filtering criteria (FC = 1.0 and FDR corrected *P*-value < 0.05). Green or red dots represent metabolites that are significantly increased or decreased after TBI, respectively. 13% of the metabolites showed a significant change between sham and TBI. Table on right shows metabolites that are significantly different as denoted by respective numbers in the figure on the left panel. **(B,C)** PLS-DA analysis of the metabolite subgroup showing distinct class separation between sham and TBI. **(D)** PLS-DA Model validation analysis and the predictive accuracy indicate good accuracy of *Q*^2^ = 0.41, 0.68 and 0.86 for the three components, respectively.

Comparison between sham and TBI showed significant changes in only 13% of the neurotransmitter metabolites. Levels of adenosine and N-acetyl aspartic acid (NAA) and adenosine decreased in TBI compared to sham (Figure 5A). However, multivariate analyses using PLS-DA (Figure 5B,C) showed distinct class separation between sham and TBI with a good accuracy of the PLS-DA model (Accuracy = 1.0, *R*^2^ value = 0.95, *Q*^2^ value = 0.86) (Figure 5D). The results indicate that neurotransmitter perturbations after TBI was dominated by NAA and adenosine changes with insignificant contribution from other neurotransmitters.

### TBI-Induced Amino Acid Metabolic Perturbations

**FIGURE 6:**
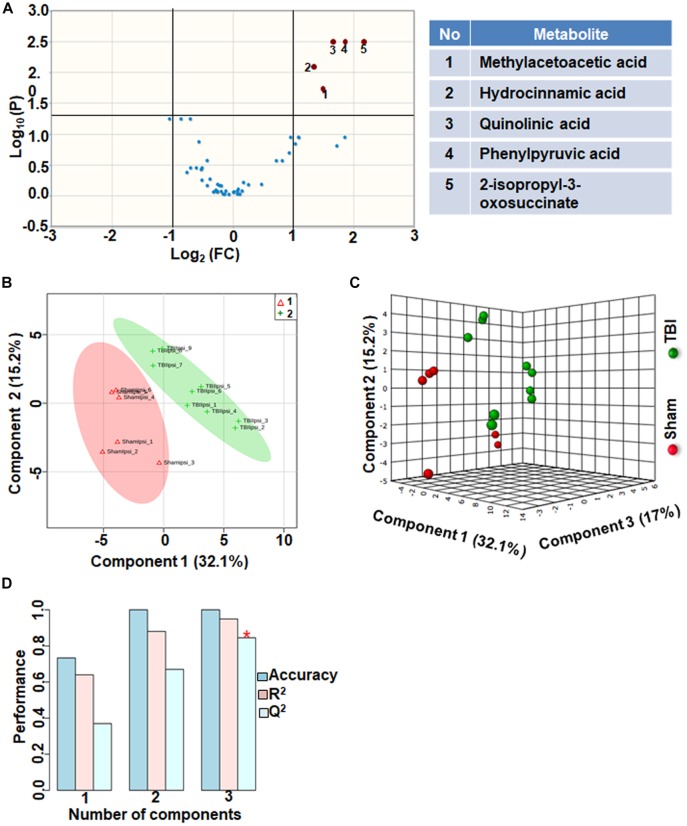
Amino acid metabolism 72 h after TBI in the developing brain. **(A)** Volcano plot showing FC on *x*-axis and FDR-adjusted *P*-values on *y*-axis. Black vertical and horizontal lines reflect the filtering criteria (FC = 1.0 and FDR corrected *P*-value < 0.05). Green or red dots represent metabolites that are significantly increased or decreased after TBI, respectively. 10% of the metabolites showed a significant change between sham and TBI. Table on right shows metabolites that are significantly different as denoted by respective numbers in the figure on the left panel. **(B,C)** PLS-DA analysis of the amino acid metabolite subgroup showing distinct class separation between sham and TBI. **(D)** PLS-DA model validation analysis and the predictive accuracy indicate high accuracy of *Q*^2^ = 0.36, 0.67 and 0.84 for the three components, respectively.

Amino acids and their related anabolic and catabolic products were grouped together as related to amino acid metabolites. TBI showed significant changes in 10% of the amino acid metabolic products, when compared to sham. A unique aspect of TBI impact on amino acid levels was that every significantly changed amino acid showed only decreases (Figure 6A). Multivariate analyses using PLS-DA (Figure 6B,C) showed distinct class separation between sham and TBI with a good accuracy of the PLS-DA model (Accuracy = 1.0, *R*^2^ value = 0.94, *Q*^2^ value = 0.84) (Figure 6D).

### TBI-Induced Alterations in Other Metabolites

**FIGURE 7:**
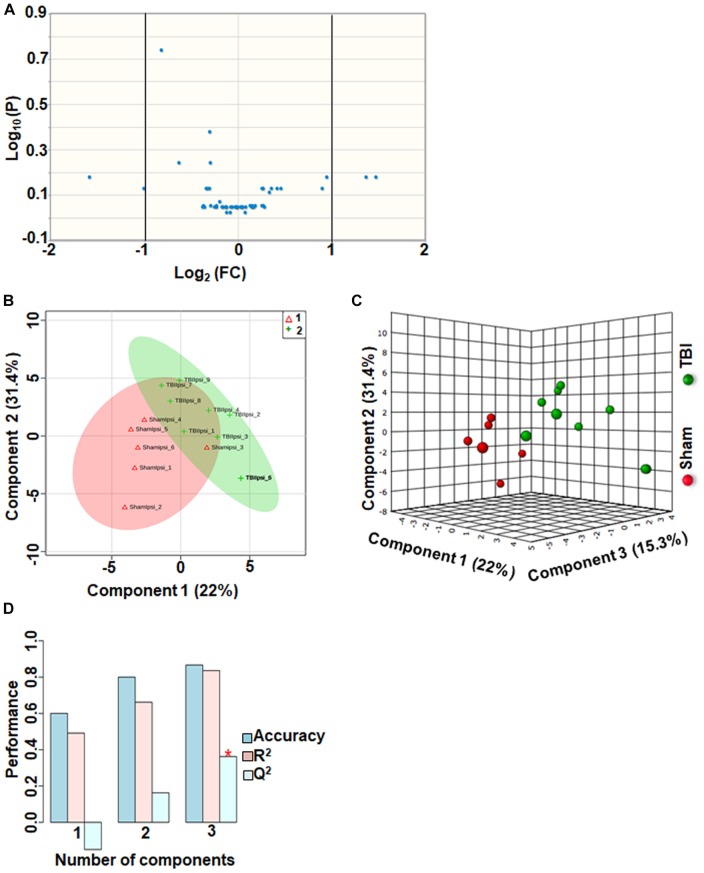
Other metabolites at 72 h after TBI in the developing brain. **(A)** Volcano plot showing FC on *x*-axis and FDR-adjusted *P*-values on *y*-axis. Black vertical and horizontal lines reflect the filtering criteria (FC = 1.0 and FDR corrected *P*-value < 0.05). None of the metabolites in this group showed a significant change between sham and TBI. **(B,C)** PLS-DA analysis of the other metabolites subgroup showed distinct classes between sham and TBI. **(D)** PLS-DA model validation analysis and the predictive accuracy was significantly low *Q*^2^ = –0.15, 0.16 and 0.36 for the three components, respectively, indicating that TBI impact was not significant.

Approximately 56 metabolites were sub-grouped as other metabolites, and none of the metabolites in this group were significantly different between sham and TBI (Figure 7A). Although multivariate analyses using PLS-DA (Figure 7B,C), showed partial class separation between sham and TBI, the accuracy of the PLS-DA model was weak from the model validation (Accuracy = 0.86, *R*^2^ value = 0.83, *Q*^2^ value = 0.36) (Figure 7D).

## Discussion

**FIGURE 8:**
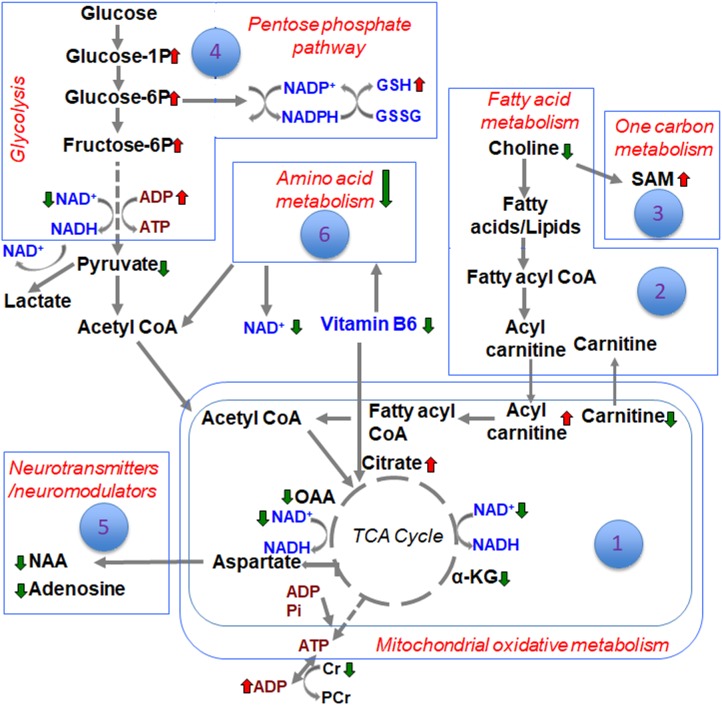
Schematic of the different biochemical pathways affected by TBI and the ranking of developmental TBI impact on each of them.

The metabolite sets determined by MSEA related to specific biochemical pathways affected by TBI in our earlier study (Chitturi et al., 2018), was similar to the consolidated biochemical profiles determined by the present study (Figure 1–6) with oxidative energy metabolism being the most impacted (Figure 1). A significant subgroup classified as other metabolites, which did not belong to any critical biochemical pathway was observed to have no significant effect after TBI (Figure 7). The following discussion describes the specificity of TBI-induced changes in the major biochemical pathways chronologically ranked based on the neurochemical impact (Figure 8).

Energy crisis develops after TBI and known to be associated with poor neurological outcomes (Ross and Soltesz, 2000; Prins et al., 2013). A strong correlation between intense ipsilateral metabolomic perturbations and neuronal cell death in the current developmental rat model indicated the involvement of multiple metabolic networks in addition to energy metabolism (Chitturi et al., 2018). These broad TBI-induced metabolic pathway disturbances led to poor ipsilateral neural viability along with dysfunctional neuronal and neurovascular activities into adolescence (Murugan et al., 2016; Parent et al., 2019). Oxidative energy metabolic depression had the highest impact on the subacute TBI pathophysiology with a *Q*^2^ = 0.89 (Figure 1). Amidst lowered energy metabolism and reduced creatine levels, ATP generation is affected and accordingly we observed increases in ADP levels after TBI (Figure 1). A significant ipsilateral decrease in N-acetyl-aspartate (NAA) levels in TBI when compared to sham (Figure 5A), indicated mitochondrial and glycolytic dysfunction dominating the several mechanisms enroute to neuronal death (Bates et al., 1996). This emphasizes the central role of bioenergetic stress at the apex, the intensity of which may predominantly steer the subacute stage secondary neurodegenerative process after TBI. The neurodegeneration reflected by NAA decrease is likely to be in selective cellular populations as secondary neurodegeneration is an ongoing process at 72 h after TBI and continues through deeper cortical layers up to 7 days in the current rat model (Lifshitz and Lisembee, 2011). Highly significant decrease in oxaloacetate (OAA) and increase in citric acid (Figure 1B), along with significant changes in α-ketoglutarate (α-KG) levels support an intensely reduced TCA cycle flux (Figure 1A). Additionally, decreased NAD levels indicated impaired rate of flow of NAD (an important co-factor) through the TCA cycle, which conforms to the decreased α-KG and OAA and increased citrate levels (Figure 1A).

Glycolysis is known to be affected after TBI due to insufficient supply of NAD (Dusick et al., 2007; Jalloh et al., 2015). However, PPP up regulation through glucose shunting toward PPP can to a certain extent maintain NAD supply and help restore TCA cycle metabolism (Bartnik et al., 2005). The specificity of glycolytic changes showing decreases in pyruvate along with accumulating glucose 1-phosphate, glucose 6-phosphate, fructose 6-phosphate and glucosamine-6-phosphate (Figure 4A), conformed a decreased glycolytic and increased oxidative phase of PPP metabolism. High impact of the glycolytic and PPP pathways to the TBI outcome observed by the PLS-DA model accuracy (Figure 4B–D), suggest a PPP- based support to TCA cycle based oxidative metabolism. Subacute stage PPP support may probably occur through restoration of the falling NAD levels. Furthermore, a significant increase in reduced form of glutathione (GSH) observed after TBI (Figure 3A) indicated an up regulated PPP, which may sustain the increased need for reducing equivalents in the subacute stage after TBI. GSH is a tripeptide known to combat oxidative stress to maintain normal cellular redox states (Winkler et al., 1986), formed by the donation of electrons from NAD(P)H to oxidized glutathione (GSSG) (Winkler et al., 1986), The decreased glycolytic and TCA cycle metabolism along with increased PPP at P31-day development indicate a prolonged bioenergetic stress, distinct from the relatively lesser intensities observed in the immature (*P* < 13) (Robertson et al., 2013) or adult TBI (Lee et al., 1999; Sheline et al., 2000; Aquilani et al., 2005; Prins, 2017).

Although relatively less impacted (*Q*^2^ = 0.72) compared to oxidative energy metabolism (*Q*^2^ = 0.89), fatty acid metabolism showed significant changes in several metabolites in the subacute stage after TBI. Choline, a principal lipid component of membrane phospholipids was significantly decreased indicating that repair of membranes and synaptic integrity may continue to be affected during the subacute stages after TBI. L-carnitine, an essential metabolite for transport of long-chain fatty acids across the mitochondrial membrane (Jones et al., 2010), showed significant decrease. Given the continued depression of oxidative metabolism, this may lead to possible consequences of metabolic encephalopathy, characterized by swollen astrocytes and enlarged mitochondria (Kimura and Amemiya, 1990). Carnitine levels are required for normal mitochondrial function, and have the ability to repair mitochondrial function and improve functional recovery after brain injury (Scafidi et al., 2010). Treatment with L-carnitine and acetyl L-carnitine has shown significant protection in the developing brain after a TBI (Ferreira and McKenna, 2017), indicating the importance of maintaining energy production through alternate means such as fatty acid oxidation. Several acyl carnitines such as L-acetyl carnitine, hexanoyl-carnitine, and propionyl-carnitine showed significant increase after TBI (Figure 2). Accumulation of acyl carnitines is an indicator of decreased mitochondrial TCA cycle flux relative to fatty acid, glucose and/or amino acid oxidation (Ribel-Madsen et al., 2016), indicative of severe mitochondrial failure and ongoing neurodegeneration in selective populations of neurons. Although accumulating acyl carnitines increase fatty acid oxidation producing acetyl CoA, it consequently increases oxygen demand in neurons, leading to increased oxygen extraction fraction (OEF) and hence cellular hypoxia (Schonfeld and Reiser, 2013). Mitochondrial populations are particularly vulnerable in the brain with high concentrations of acyl carnitines and regional hypoxia, known to worsen TBI outcomes as observed in humans (Jeremitsky et al., 2003; Chi et al., 2006; Spaite et al., 2017; Vella et al., 2017). Significant decrease in the levels of 3-Hydroxyhexadecanoic acid (beta oxidation product of saturated fatty acid; palmitic acid) indicated increased oxidation of fatty acids during the subacute stage after TBI (Figure 2A). Medium chain fatty acid, caprylic acid/octanoic acid levels were also decreased after TBI. Caprylic acid in the brain serves as a ketogenic compound where ketones from caprylic acid generate necessary substrates for metabolically perturbed mitochondria in order to sustain energy metabolism (Thaipisuttikul and Galvin, 2012). Further studies are required to ascertain if the subacute ketogenic shift in brain metabolism after TBI may be an intrinsic repair mechanism.

One carbon metabolism involving folate and methionine metabolism, is required for SAMe synthesis, a major DNA methylating agent. One carbon metabolism is also critical for protein translation, sulfhydration, nucleotide, amino acid and lipid head group metabolism (Locasale, 2013). TBI increased SAM and glutathione levels (Figure 3A), impaired SAM levels have been reported in chronic neurodegenerative disease (Coppede, 2010). TBI also resulted in significant reduction in two forms of vitamin B6- pyridoxine and pyridoxamine, and its catabolic product 4-pyridoxic acid (Figure 3A). As B vitamins are essential co-enzymes for TCA cycle, electron transport chain and glycolysis (Kennedy, 2016), deficiency in any one form of vitamin B may exacerbate the harmful effects of TBI emanating from reduced brain energy metabolism. Vitamin B6 is also essential for the catabolism and transamination of amino acids (Hwang et al., 2007). A high impact of the one carbon/folate metabolic pathway to TBI biochemical changes (*Q*^2^ value = 0.82; Figure 3B–D), combined with therapeutic evidence of improved TBI outcomes after administration of pyridoxine 30 min after a unilateral TBI (Kuypers and Hoane, 2010), highlight the importance of maintaining vitamin B6 metabolism after TBI.

Amino acid metabolism was significantly impacted after TBI, (*Q*^2^ value = 0.84; Figure 6B–D). Specificity of changes showed a decrease in methylacetoacetic acid, a beta-keto acid and by product of isoleucine metabolism. Branched amino acids (BCAAs) are both glucogenic (valine, isoleucine) and ketogenic (leucine, isoleucine) and play an important role in energy production and neurotransmitter synthesis. Phenylalanine metabolism was significantly affected as observed by significant decreases in phenyl pyruvic acid and hydrocinnamic acid (Figure 6A). Alterations in phenylalanine metabolism results in phenylketonuria, a detrimental condition to normal brain development and cognitive maturation (Schuck et al., 2015). Hence perturbed phenylalanine metabolism at the subacute stage after TBI may affect ongoing developmental plasticity. Quinolic acid, a metabolite of amino acid tryptophan also decreased significantly after TBI. Tryptophan metabolism is critical for normal brain function through the production of nicotinic acid, a precursor of the important co-factor NAD, via metabolism of quinolic acid (Moroni, 1999). NAD levels which significantly decreased after TBI (Figure 1), maybe influenced by decreases in the levels of quinolic acid. Hence the impact of amino acid metabolic changes together with the impact of energetic stress may provide a basis to interpret long-term changes in pediatric TBI outcomes.

An important observation in the current study was the large subgroup of remaining metabolites (56 out of a total of 190) classified as “other metabolites” having no significant impact after TBI (*Q*^2^ = 0.36). Specificity of changes also did not indicate any significantly perturbed metabolites (Figure 7). A greater than 2-fold increase in gluconic acid was, however, observed. While it is known that gluconic acid is produced from glucose, further studies are required to determine the exact significance of its increase after TBI.

## Conclusion

Differences between sham and TBI over the consolidated biochemical pathways as summarized in the schematic (Figure 8), indicated a broad TBI-induced biochemical shift in the developing brain at the subacute stage of 72 h after injury. Highest impact on brain bioenergetics with depressed TCA cycle flux and a glycolytic shift toward PPP was observed, probably as a response to maintain NAD supply and help restore TCA cycle metabolism. Several other pathways such as fatty acid, amino acid and neurotransmitter metabolism could be relatively ranked. While the current study results represent the subacute scenario in developmental TBI, it provides a consolidated biochemical template to characterize a variety of experimental TBI in animal models.

## Ethics Statement

This study was carried out in accordance with the recommendations of ARRIVE guidelines. All experimental procedures were approved by the Institutional Animal Care and Use Committee of Rutgers Biomedical and Health Sciences, New Jersey Medical School.

## Author Contributions

SK conceived and designed the study. JC, YL, and SK performed the experiments. JC analyzed the data, VS contributed to significant laboratory resources and expertise for the animal model of Traumatic Brain Injury, JC and SK wrote the first draft of the manuscript. All authors read and approved the submitted version of the manuscript.

## Conflict of Interest Statement

The authors declare that the research was conducted in the absence of any commercial or financial relationships that could be construed as a potential conflict of interest.

## Footnotes

1 http://www.metaboanalyst.ca
