# Supplementary material for: Consolidated Biochemical Profile of Subacute Stage Traumatic Brain Injury in Early Development
Source: Front Neurosci. 2019 May 3;13:431. doi: 10.3389/fnins.2019.00431 (PMC6509949; doi:10.3389/fnins.2019.00431)
Supplement: Supplementary file 1 [file Table_1.DOCX]

| **Oxidative Energy and Mitochondrial Metabolites**  **Table S1.** List of brain metabolites identified and characterized. Metabolites were sub-grouped based on critical pathways implicated in the rat model of mild/moderate developmental TBI from previous studies. | |
| --- | --- |
|  |  |
| 1 | Citric acid |
| 2 | Maleic acid |
| 3 | Oxaloacetic acid |
| 4 | Oxoglutaric acid |
| 5 | Succinic acid |
| 6 | Trans-Aconitic acid |
| 7 | Itaconic acid |
| 8 | Inosinic acid |
| 9 | Guanosine diphosphate |
| 10 | Adenosine monophosphate (AMP) |
| 11 | ADP |
| 12 | NAD |
| 13 | NADP |
| 14 | Betaine aldehyde |
| 15 | 2-Ketobutyric acid |
| 16 | Glycolic acid |
| 17 | Glyoxylic acid |
| 18 | Creatine |
| **Glycolysis and Pentose Phosphate Pathway** | |
|  |  |
| 19 | Glucose 1-phosphate |
| 20 | Glucose 6-phosphate |
| 21 | Fructose 6-phosphate |
| 22 | Deoxyribose 5-phosphate |
| 23 | Dihydroxyacetone phosphate |
| 24 | Glycerol 3-phosphate |
| 25 | D-Sedoheptulose 7-phosphate |
| 26 | Phosphoenolpyruvic acid |
| 27 | Pyruvic acid |
| 28 | L-Lactic acid |
| 29 | D-Arabitol |
| 30 | D-Fructose |
| 31 | D-Glucose |
| 32 | D-Ribose |
| 33 | D-Ribose 5-phosphate |
| 34 | Acetylphosphate |
| 35 | Glucosamine 6-phosphate |
| **Fatty Acid Metabolites** | |
|  |  |
| 36 | Choline |
| 37 | Citicoline |
| 38 | Glycerophosphocholine |
| 39 | Hexanoylcarnitine |
| 40 | L-Acetylcarnitine |
| 41 | (R)-3-Hydroxydecanoic acid |
| 42 | (R)-lipoic acid |
| 43 | 2-Hydroxycaproic acid |
| 44 | 2-Hydroxyvaleric acid |
| 45 | 3-hydroxyhexadecanoic acid |
| 46 | 3-Hydroxymethylglutaric acid |
| 47 | Caprylic acid |
| 48 | CDP-Ethanolamine |
| 49 | DL-2-Aminooctanoic acid |
| 50 | Phosphorylcholine |
| 51 | L-Carnitine |
| 52 | D-2-Hydroxyglutaric acid |
| 53 | Propionylcarnitine |
| 54 | Phenylacetylglycine |
| **Amino acids and related metabolites** | |
|  |  |
| 55 | Argininosuccinic acid |
| 56 | Asymmetric dimethylarginine |
| 57 | Citrulline |
| 58 | L-Alanine |
| 59 | L-Arginine |
| 60 | L-Asparagine |
| 61 | L-Glutamine |
| 62 | L-Histidine |
| 63 | L-Histidinol |
| 64 | L-Isoleucine |
| 65 | L-Kynurenine |
| 66 | L-Leucine |
| 67 | L-Lysine |
| 68 | L-Methionine |
| 69 | L-Phenylalanine |
| 70 | L-Proline |
| 71 | L-Serine |
| 72 | L-Tryptophan |
| 73 | L-Tyrosine |
| 74 | L-Valine |
| 75 | L-Threonine |
| 76 | Threonic acid |
| 77 | N-Acetylglutamic acid |
| 78 | N-Acetylglutamine |
| 79 | Phenyllactic acid |
| 80 | Phenylpyruvic acid |
| 81 | Pyroglutamic acid |
| 82 | 1-Methylhistidine |
| 83 | Carnosine |
| 84 | N6-Acetyl-L-lysine |
| 85 | Ornithine |
| 86 | Pipecolic acid |
| 87 | Aminoadipic acid |
| 88 | Guanidoacetic acid |
| 89 | Hydrocinnamic acid |
| 90 | Indolelactic acid |
| 91 | Kynurenic acid |
| 92 | N-Acetyl-L-alanine |
| 93 | N-Acetyl-L-methionine |
| 94 | N-Acetylasparagine |
| 95 | N-Acetylornithine |
| 96 | N-Formyl-L-methionine |
| 97 | L-Dihydroorotic acid |
| 98 | Quinolinic acid |
| 99 | Urocanic acid |
| 100 | 2-Isopropyl-3-oxosuccinate |
| 101 | Methylacetoacetic acid |
| 102 | 2-Ketohexanoic acid |
| 103 | Isovalerylglycine |
| 104 | Nicotinic acid |
| 105 | 2-Isopropylmalic acid |
| **Neurotransmitters and Neuromodulators (Polyamines)** | |
|  |  |
| 106 | Acetylcholine |
| 107 | L-Aspartic acid |
| 108 | L-Glutamic acid |
| 109 | N-Acetylaspartylglutamic acid |
| 110 | N-Acetyl-L-aspartic acid |
| 111 | 4-Acetamidobutanoic acid |
| 112 | 4-Hydroxybutyric acid |
| 113 | 5-Methoxytryptophan |
| 114 | Putrescine |
| 115 | N-Acetylputrescine |
| 116 | Spermidine |
| 117 | 5'-Methylthioadenosine |
| 118 | Adenosine |
| 119 | Hypotaurine |
| 120 | Taurine |
| **One Carbon/Folate Metabolism** | |
|  |  |
| 121 | Dimethylglycine |
| 122 | S-Adenosylhomocysteine |
| 123 | S-Adenosylmethionine |
| 124 | Glycine |
| 125 | Betaine |
| 126 | L-Cystathionine |
| 127 | Acetylcysteine |
| 128 | Oxidized glutathione |
| 129 | Glutathione |
| 130 | 5-Methyltetrahydrofolic acid |
| 131 | Pyridoxamine (Vitamin B6) |
| 132 | Pyridoxine (Vitamin B6) |
| 133 | 4-Pyridoxic acid |
| 134 | Methionine sulfoxide |
| **Other Metabolites** | |
|  |  |
| 135 | 1-Methyladenosine |
| 136 | 1-Methylnicotinamide |
| 137 | 2'-Deoxyguanosine 5'-monophosphate |
| 138 | 2-Furoic acid |
| 139 | 2-Pyrocatechuic acid |
| 140 | 4-Hydroxybenzaldehyde |
| 141 | Adenine |
| 142 | ADP-glucose |
| 143 | Allantoin |
| 144 | Arecoline |
| 145 | Ascorbic acid |
| 146 | Beta-D-Glucopyranuronic acid |
| 147 | Creatinine |
| 148 | Cytidine |
| 149 | Cytidine monophosphate |
| 150 | Cytosine |
| 151 | Deoxyadenosine |
| 152 | Deoxyuridine |
| 153 | Ecgonine |
| 154 | GDP-glucose |
| 155 | Gluconic acid |
| 156 | Gluconolactone |
| 157 | Guanine |
| 158 | Guanosine |
| 159 | Hippuric acid |
| 160 | Hypoxanthine |
| 161 | Imidazoleacetic acid |
| 162 | Imidazolelactic acid |
| 163 | Indole-3-carboxylic acid |
| 164 | Inosine |
| 165 | Insitol |
| 166 | Malic acid |
| 167 | Mannitol |
| 168 | Methylimidazoleacetic acid |
| 169 | N-Acetyl-D-glucosamine |
| 170 | N-Acetyl-glucosamine 1-phosphate |
| 171 | Niacinamide |
| 172 | Orotic acid |
| 173 | Orotidine |
| 174 | Pantothenic acid |
| 175 | Proline betaine |
| 176 | Salicylic acid |
| 177 | Thiamine |
| 178 | Thiamine monophosphate |
| 179 | Thymidine |
| 180 | Uracil |
| 181 | Ureidosuccinic acid |
| 182 | Uric acid |
| 183 | Uridine |
| 184 | Uridine 5'-monophosphate |
| 185 | Uridine diphosphate glucuronic acid |
| 186 | Uridine diphosphategalactose |
| 187 | Uridine diphosphate-N-acetylglucosamine |
| 188 | Vanillin |
| 189 | Xanthine |
| 190 | Xanthosine |
